# Engendering healthy masculinities to prevent sexual violence: Rationale for and design of the Manhood 2.0 trial

**DOI:** 10.1016/j.cct.2018.05.017

**Published:** 2018-05-23

**Authors:** Kaleab Z. Abebe, Kelley A. Jones, Alison J. Culyba, Nayck B. Feliz, Heather Anderson, Irving Torres, Sarah Zelazny, Patricia Bamwine, Adwoa Boateng, Benjamin Cirba, Autumn Detchon, Danielle Devine, Zoe Feinstein, Justin Macak, Michael Massof, Summer Miller-Walfish, Sarah Elizabeth Morrow, Paul Mulbah, Zabi Mulwa, Taylor Paglisotti, Lisa Ripper, Katie A. Ports, Jennifer L. Matjasko, Aapta Garg, Jane Kato-Wallace, Julie Pulerwitz, Elizabeth Miller

**Affiliations:** a Division of General Internal Medicine, University of Pittsburgh School of Medicine, 200 Meyran Ave., Suite 300, Pittsburgh, PA 15213, USA; b Division of Adolescent and Young Adult Medicine, Children’s Hospital of Pittsburgh of UPMC, Department of Pediatrics, University of Pittsburgh School of Medicine, 3420 Fifth Ave, Pittsburgh, PA 15213, USA; c Division of Violence Prevention, National Center for Injury Prevention and Control, Centers for Disease Control and Prevention, 4770 Buford Highway, Atlanta, GA 30341, USA; d Promundo-US, 1367 Connecticut Avenue, NW, Suite #310, Washington, DC 20036, USA; e Population Council, 4301 Connecticut Avenue, NW, Suite 280, Washington, DC 20008, USA

**Keywords:** Teen dating violence, Adolescent relationship abuse, Sexual violence, Gender violence, Sexual harassment, Adolescent health

## Abstract

Violence against women and girls is an important global health concern. Numerous health organizations highlight engaging men and boys in preventing violence against women as a potentially impactful public health prevention strategy. Adapted from an international setting for use in the US, “Manhood 2.0” is a “gender transformative” program that involves challenging harmful gender and sexuality norms that foster violence against women while promoting bystander intervention (i.e., giving boys skills to interrupt abusive behaviors they witness among peers) to reduce the perpetration of sexual violence (SV) and adolescent relationship abuse (ARA). Manhood 2.0 is being rigorously evaluated in a community-based cluster-randomized trial in 21 lower resource Pittsburgh neighborhoods with 866 adolescent males ages 13–19. The comparison intervention is a job readiness training program which focuses on the skills needed to prepare youth for entering the workforce, including goal setting, accountability, resume building, and interview preparation. This study will provide urgently needed information about the effectiveness of a gender transformative program, which combines healthy sexuality education, gender norms change, and bystander skills to interrupt peers’ disrespectful and harmful behaviors to reduce SV/ARA perpetration among adolescent males. In this manuscript, we outline the rationale for and evaluation design of Manhood 2.0.

## 1. Introduction

### 1.1. Background and rationale

Sexual violence (SV) and intimate partner violence affect at least one in three women in the world [1] including in the United States [2]. Among adolescents in the US, non-partner SV often co-occurs with adolescent relationship abuse (ARA; physical, sexual, or emotional abuse by a partner) victimization [3], and such experiences are associated with poor health, including suicidality, depression, substance use, unintended pregnancy, and sexually transmitted infections (STIs) [4–13]. Perpetration of SV/ARA is associated with multiple individual and contextual factors, including exposure to adverse childhood experiences, poor conflict resolution and relationship skills, and norms that condone violence perpetration [14]. Prevention entails modifying potential perpetrator behaviors, which in turn requires attention to both individual attitudes and the normative peer context. [14]

This study addresses SV and ARA perpetrated against adolescent females as a gendered problem, based on multiple studies demonstrating the relationship between males’ gender inequitable practice (attitudes and behaviors that degrade women and promote ‘rigid masculinity’) and SV/ARA perpetration by adolescent males [15–27]. Gender inequitable practice is associated with poor health outcomes for men (including HIV infection) and increased violence victimization and poor outcomes for women [22,28–30]. Health interventions that focus on promoting gender equity demonstrably reduce violence and substance use, increase condom use, decrease transactional sex, and increase communication between couples. [31–39]

As SV/ARA perpetration often emerges in the context of male peers who demonstrate negative attitudes toward females, endorse bias-based prejudices regarding homosexuality and condone abuse perpetration [40–47], prevention requires addressing potential perpetrator attitudes and behaviors as well as the gendered peer environment in which they are embedded. Perceived peer tolerance for SV/ARA may promote individual likelihood of these behaviors, and may reduce comfort and ability to intervene when faced with negative behaviors among peers, contributing to a social climate that enables such behavior [42]. Many violence prevention programs focused on social norms change employ a bystander behavior approach, in which individuals are taught skills to respond with active intervention in SV/ARA rather than with apathy or tolerance [48,49]. This study draws on building bystander intervention skills combined with evidence from international settings that demonstrated the effectiveness of encouraging critical analysis of gender norms, challenging homophobia and gender-based harassment, and building skills both to critically question harmful masculine norms and to employ more equitable behaviors.

The literature on adolescent sexual health promotion also underscores the need for skills building that includes an emphasis on respect, communication about pregnancy and STI and HIV/AIDS prevention, condom negotiation, sexual consent, and learning about reproductive and sexual coercion [50–52]. The most effective sexual health interventions also address gender and power [53]. Integration of open, in-depth discussions about respectful sexual behaviors that also address homophobia and rigid masculinity norms may simultaneously reduce SV/ARA perpetration and improve sexual health. In international settings, sexual health promotion programs that incorporate changing cultural norms around masculinity (i.e., “gender transformative” programs), focused on older adolescents and young adults, have demonstrated significant positive shifts in gender attitudes as well as increased use of condoms and decreased reporting of men’s use of violence towards an intimate partner [29,32,43,54,55]. This is the first study to test the effectiveness of a community-based program for adolescent males that combines healthy sexuality skills, gender norms change, and bystander skills to interrupt peers’ disrespectful and harmful behaviors to prevent SV/ARA perpetration among adolescent males.

## 2. Methods

### 2.1. Adaptation of an international gender transformative curriculum

**Fig. 1.**
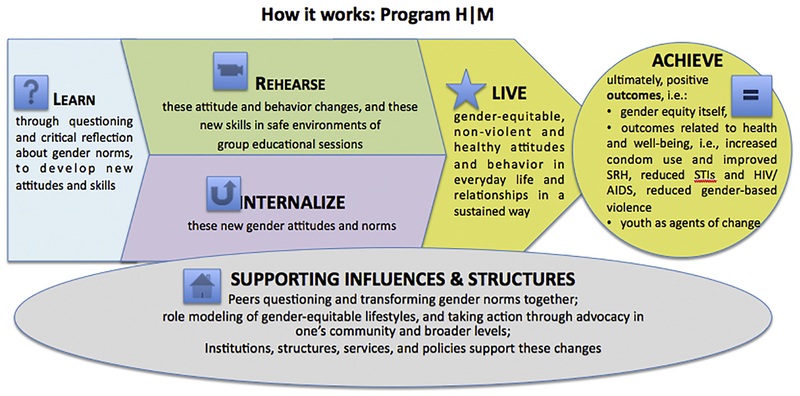
Conceptual model for Program H.

The intervention to be tested in this study – titled “Manhood 2.0” – is the first U.S. adaptation of the well-established Program Η (“H” stands for “homem” meaning “man” in Portuguese), a gender-transformative curriculum tailored for young men (https://promundoglobal.org/programs/program-h/). Developed in Brazil by Promundo (a global gender equality and violence prevention organization) and their partners in global health, Program H is an integrated curriculum and community outreach model (see: Fig. 1) to engage adolescent and adult men in health promotion, gender equality, and gender-based violence prevention that has been implemented in 29 countries. The evaluation studies conducted to date in global settings have found promising changes in attitudes that support gender-based violence and in some settings to lead to reductions in young and adult men’s reported use of violence. Based on these results, Program H has been acknowledged by PAHO, UNICEF, UNFPA, UNDP, the World Bank and the Brazilian Ministry of External Relations as a best practice in promoting gender equality.

Program H was identified by the PI as a promising program to adapt for efforts to prevent SV/ARA in the U.S. The key adaptations of Program H to create Manhood 2.0 include additional discussions of social media use, internet pornography, deeper explorations of inter-sectionality using visual art (examining the unique experiences of racism and marginalization experienced by young African-American men in the United States, examining white privilege, male privilege), female-controlled contraception (including long-acting reversible contraception [LARCs]), and practicing bystander intervention skills.

#### 2.1.1. Theoretical and empirical basis for the intervention

There are several theoretical and empirical bases for the Manhood 2.0 intervention on SV/ARA perpetration, including:

##### 2.1.1.1. A program that integrates gender norms change, education about healthy sexuality, and promotion of positive bystander intervention behaviors is likely to address several modifiable risk factors related to SV/ARA perpetration.

From the evidence emerging from the epidemiology of SV/ARA and studies of current SV/ARA prevention programs, SV/ARA perpetration prevention requires integration of several core intervention components that are theoretically and empirically grounded [14,56–59]. Consistent with Social Norms Theory [42,60] and Theory of Reasoned Action combined with the Theory of Gender and Power [61], the Manhood 2.0 program trains prevention educators to facilitate discussions with adolescent males that: 1) promote gender equitable attitudes, 2) encourages adolescent males to reflect on how gender norms and power dynamics influence behaviors related to violence and sexual behaviors 3) educates adolescent males in healthy sexuality skills to increase sexual communication, consent, and recognition of sexual coercion, and 3) encourages positive bystander intervention when witnessing violent and inequitable behaviors among peers. By encouraging critical reflection and challenging harmful and violent and inequitable behaviors in the context of heterosexual relationships, this intervention aims to address the parts of youth socialization that endorse norms, attitudes, and behaviors that facilitate violence and unhealthy behaviors. In doing so, the program aims to promote critical transformation of these norms towards gender equity.

Shifting gender norms, reducing homophobic attitudes, educating about sexuality and sexual consent, and developing positive bystander intervention skills influence not only individual attitudes and behaviors, but also the peer and social context in which these youth are embedded. A program that integrates these areas (gender norms change around rigid masculinities, reflection on power dynamics, education about healthy sexuality, and positive bystander intervention) has not yet been rigorously evaluated in the U.S.

##### 2.1.1.2. Community-based SV/ARA prevention programs which are flexible around how and when curriculum is delivered and which do not rely on school district approvals for implementation are needed.

Some school-based teen pregnancy and HIV prevention education efforts in the U.S. have emphasized abstinence and facts about contraception, condom use, and STIs [62,63]. Simultaneously, few pregnancy and HIV prevention programs address SV/ARA, sexual consent, condom negotiation, and gender norms. Whereas school-based classroom instruction is often broken into one to two hour segments [64–66], implementation in more informal community settings allows for lengthier and deeper conversations about sexuality and violence over the course of several days that is likely to increase interactions, questions, and personal reflections among youth [67].

##### 2.1.1.3. Youth development-focused community programs engage adolescent males living in socially disadvantaged neighborhoods through a range of modalities including community-based athletics, after school programs, employment programs, and truancy programs.

The population for this study is adolescent males ages 13–19 (primary focus is high school-age youth) living in the socially disadvantaged, primarily African American neighborhoods of Pittsburgh. The rationale for focusing on primarily African American youth in lower resource neighborhoods is two-fold.

First, racial/ethnic disparities in health in Pittsburgh are stark. The county has the second highest rate of teen birth to African American adolescent females in the state [68], a prevalence of STIs among African American adolescent females that is twice the national average [69,70], while firearm-related injuries and death disproportionately impact both African American males and females [71,72]. Exposures to community violence, as well as the social context for unintended pregnancy and STIs, are closely linked to increased vulnerabilities for sexual violence [45,47,73]. Our research and others have underscored the multiple ways that poverty increases these vulnerabilities including through economic dependency, sexual exploitation, drug trade, survival sex, and gang affiliation [73–76]. Prevention efforts in communities with high prevalence of poverty, violence, and poor health outcomes are needed, informed by the social and economic justice frameworks that are the foundational principles among the community partners and stakeholders participating in this project.

Second, the prevention educators working within these youth-serving agencies are from the communities in which they are working, and are highly trusted and respected. Many also oversee informal athletic programs, after-school programs, and school-based prevention education, allowing them to work across multiple social settings. Thus, these community programs and facilitators are well positioned to recruit and retain a heterogeneous sample of adolescent males and their peers through these existing social networks. In line with core principles for prevention programs, as recommended by Nation [67], these prevention educators are likely to be able to connect with youth in meaningful and sustainable ways.

##### 2.1.1.4. Sustainable, scalable community programs to prevent SV are needed.

We have too few evidence-based SV/ARA prevention programs in the U.S. focused on adolescents that can be delivered by community members/youth agency staff without extensive training [64–66]. We have only one evidence-based SV/ARA prevention program focused on adolescent males and that is in the context of school-based athletics only [37,77]. No evidence-based SV/ARA programs for youth take place outside of the classroom or school-based athletics setting. This study will advance scientific knowledge about SV/ARA perpetration prevention (with an emphasis on primary prevention) and address these gaps in the existing evidence base. This research will provide urgently needed information about the relevance of an innovative community-based SV/ARA prevention program adapted from international prevention efforts for implementation with adolescent males in community-based settings.

The intervention involves an 18 h curriculum divided into six 3h sessions delivered once or twice a week (generally over 3 to 6 week time period). This design builds on primary prevention principles that emphasize a comprehensive, theory-driven approach, sociocultural relevance, well-trained staff, opportunity for building positive relationships with youth, sufficient dosage (through repeated exposure to content), and youth participation balanced with feasibility and cost of implementation approaches [67,78]. Program implementation relies on the youth development infrastructure and community-based networks already in place at participating YMCAs, Urban League, and other youth development organizations in Pittsburgh, including the ability to reach diverse adolescent males with the assistance of other youth-serving community agencies including schools, libraries, and churches. Multiple stakeholders at the local, regional, national, and global level are involved to ensure the program is relevant, easy to implement, and replicable, thus if found to be effective, could be widely disseminated as a promising prevention program.

### 2.2. Objectives

This cluster-randomized community-based intervention will examine the effectiveness of a program for the primary prevention of SV/ARA titled “Manhood 2.0.” This program seeks to alter gender norms that foster SV/ARA perpetration, while promoting bystander intervention (i.e., giving boys skills to interrupt disrespectful and abusive behaviors they witness among peers) and respectful sexual behaviors, to reduce SV/ARA perpetration.

The primary objective of this study is to test the effectiveness of Manhood 2.0 compared to a job skills development curriculum on 1) reductions in self-reported perpetration of SV and ARA (*Primary Outcome)* toward females and 2) increased positive bystander intervention behaviors (*Secondary outcome*). Intermediate outcomes include increased condom self-efficacy; contraceptive use attitudes; increased recognition of abusive behaviors; increased gender-equitable attitudes; and increased intentions to intervene with peers.

### 2.3. Trial design

**Fig. 2.**
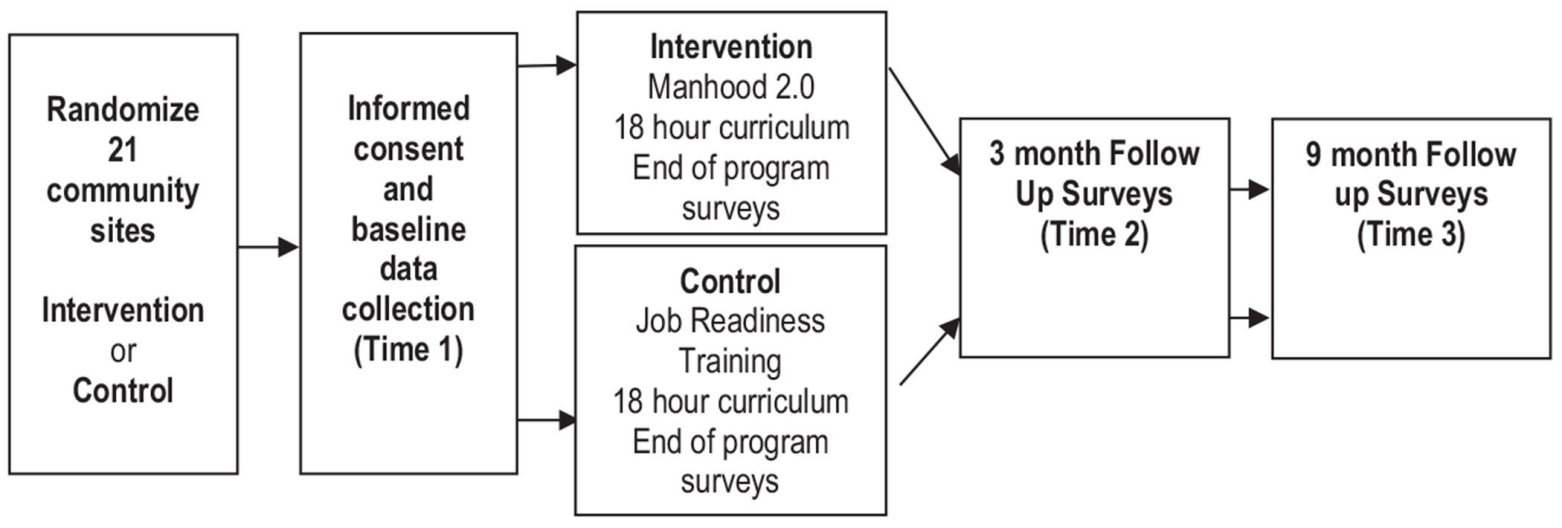
Manhood 2.0: engendering healthy masculinities – study flow.

This study design involves a two-arm cluster-randomized-controlled trial conducted with adolescent males ages 13–19 recruited from youthserving community agencies in Pittsburgh, PA. Twenty-one clusters from 20 neighborhoods were randomly allocated to the intervention or control arm. Participants (n = 866) complete surveys prior to program implementation (baseline) and immediately following the program (end of program, EOP). Follow-up surveys are collected 3 months (T2) and 9 months (T3) after end of program. Baseline surveys are completed in-person using tablets to complete the survey online; EOP, T2, and T3 are also completed in-person on a tablet or remotely using survey links that are texted or emailed to participants using contact information provided with recruitment. Retention is facilitated by collecting detailed contact information and offering incentives for survey completion ($50 total for baseline, feedback surveys throughout the program, and EOP; $30 for T2; $50 for T3) (see Fig. 2 for study flow).

### 2.4. Participants, interventions, and outcomes

#### 2.4.1. Study setting and site eligibility criteria

This study involves 20 neighborhoods and 21 clusters in the Pittsburgh area; within each neighborhood, one to four different community partner organizations (referred to as “community partners” here) participated. Neighborhoods were recruited by identifying a potential community partner who could host the program and were willing to be randomized to receive the intervention or control programming. These community partners included youth-serving organizations, YMCA, Urban League, faith-based organizations, and libraries. Additionally, we partnered with the county’s community intensive surveillance program, a diversion program for youth involved in the juvenile justice system.

Neighborhoods were identified based on having sites where the YMCA, Urban League, or other youth serving partners had existing programs and which were considered lower income communities based on census information and school district data. Asset maps were created for each neighborhood with the goal of identifying community champions and youth-relevant resources to support this community-based project. The strong partnerships established with key stakeholders in each of these sites (including site coordinators, facilitators, and community members) facilitated recruitment and retention as described below.

Among the 20 participating neighborhoods, the proportion of students attending public high schools in those neighborhoods considered economically disadvantaged ranges from 32 to 100%; the high school graduation rate for those same school districts ranged from 63 to 97%. Each of these neighborhoods struggle with poverty, school ‘push-out’ (disciplinary actions that push youth, especially African American boys, out of the regular school system), and among the highest rates of gang and gun violence in the county (see Table 1 for neighborhood characteristics). These characteristics were compared by treatment arm using Kolmogorov-Smirnov two-sample exact tests due to their nonnormal distributions. Neighborhood characteristics did not vary between the intervention and control arm neighborhoods.

Within the 20 participating neighborhoods, there were a total of 40 sites/community partners/locations approached and 38 agreed to participate. The composition of the participating sites included 11 places of worship, 20 community centers, 2 public libraries, and 5 juvenile justice community intensive surveillance program centers. One site (cluster) was located at the downtown Urban League office and consisted of youth involved in an African American young men’s leadership group who came from several different neighborhoods and schools in the Pittsburgh area.

#### 2.4.2. Participant eligibility criteria

Manhood 2.0 was designed for high school age youth for implementation in community-based settings. Eligible youth were between the ages of 13 to 19, who identified themselves as male, were residents in the neighborhood where the site was located, and were willing to participate in an 18 h gender-specific program. Youth were allowed to participate in the program (intervention or control) without participating in the research.

### 2.5. Experimental and control arms

Program Delivery: Both experimental and control arm interventions involved 18 h of curriculum, generally spread out over 3 to 6 week periods. The program was delivered with some variation in schedules to meet the needs of community partners and participating youth. Such configurations included but were not limited to: three 6-h sessions spanning three weekdays during the summer (for job skills training only), nine 2-h sessions held twice per week on weekday evenings, and six 3-h sessions held once or twice per week on weekday evenings or Saturday afternoons (which was the preferred and most commonly used design).

Fidelity to intervention: For both intervention and control arms, research assistants were present at every session to track attendance, facilitate program implementation logistics (such as ordering food), and to complete detailed fidelity forms that assessed facilitators’ ability to deliver the content of the program as intended.

### 2.6. Manhood 2.0 (experimental arm)

In prior implementations of the Program H curriculum in international settings, we found that intensive sessions spread over a period of several weeks results in greater uptake by youth and is more feasible in community settings (rather than trying to schedule a 3 day consecutive, overnight event that incurs significantly more resources) [29,54].

Program Content: Manhood 2.0 guides youth to explore and reflect upon social constructions of masculinity, describe healthy relationships, discuss healthy sexual behaviors, identify coercive and disrespectful behaviors, and practice skills to intervene when witnessing peers’ disrespectful and harmful behaviors, with repeated reflection on gender norms throughout these sessions. The curriculum involved three main topic areas. The first focuses on the theme of gender, masculinity and power, allowing the young men to actively reflect on the messages and expectations that they have received from society about manhood and gender norms. The second topic focuses on the theme of violence. This includes several components: an exploration of the various forms of violence, its impact on communities, and the role that masculinity may contribute; identifying healthy versus unhealthy romantic relationships; sexual consent and decision making; and bystander interventions when witnessing abusive behavior. The final topic area focuses on sexual and reproductive health, which includes providing information about sexual health and contraception, condom and contraceptive demonstrations, tying health behaviors and access to health facilities to conceptions of masculinity, and opportunities to ask medical professionals sexual health-related questions.

### 2.7. Job skills curriculum (control arm)

The job skills readiness program was developed and tested, and is widely used throughout the county, called “Jump Start Success Work Readiness and Career Exploration Training” (http://www.youthworksinc.org/jumpstart_success/index.html). The sessions were set up to mimic the timing for the intervention curriculum (18 h curriculum).

Program Content: The curriculum covers topics from career options and goal setting to interviewing skills. The entire curriculum involves 9 modules. For the purposes of this study, the facilitators focused on the first 6 modules (to mimic Manhood 2.0 structure) with an emphasis on goal setting, future orientation, learning about building a resume, interviewing skills, and workplace expectations.

### 2.8. Training of facilitators for control and intervention

The initial training for Manhood 2.0 occurred over 3 days to develop a core group of facilitators for the study. The first 2 days involved understanding the program’s methodology, learning more about the activities and the opportunity to practice activities for feedback from their colleagues; the final day involved a pilot with boys from a school district not involved in the study who provided feedback on the curriculum for further refinement. Because *Jump Start* is an established program with an experienced facilitator, an initial training for the facilitator of the control group was not necessary.

Subsequent trainings for both the control and experimental programs were primarily comprised of one on one and/or small group mentorships, using an apprenticeship model, where the facilitator(s) shadowed and worked alongside an experienced lead facilitator at a site, for the entire 18-h curriculum. This model enabled facilitators to pair with a lead facilitator and work their way from observing a discussion and/or session to co-facilitating to finally leading a complete session on their own, through an iterative process encompassing a series of check-ins, evaluations, and feedback. During the observation process, facilitators were encouraged to experience and examine all elements of the program, particularly activity and session flow and timing and the dynamic between facilitators and participants and amongst participants themselves. In addition, during the observation period, facilitators were required to review and become familiar with the curriculum. Depending on experience and comfort level, facilitators were welcomed to engage in discussion. Facilitators were deemed ready to lead a session if they had participated as a co-facilitator, were observed leading each activity, and if the feedback forms from youth and fidelity checks conducted by research assistants consistently showed adherence to program content. This approach to training community-based facilitators creates a longer-term sustainability plan, with lead facilitators training newly interested facilitators from their community.

After each session, the facilitator would reconvene with the lead facilitator to debrief and examine implementation. Participant engagement and behavior, program delivery and timing, and learning objectives were priority topics addressed during debriefing. These elements were also captured on fidelity forms completed by research assistants during each program session. Post-session development also included biweekly check-ins with project coordinators to analyze feedback and fidelity forms. These meetings ensured maintenance of fidelity and program goals, in a timely fashion. If facilitators needed to improve on the delivery of a certain activity or topic or if there were issues with engagement, immediate action was taken to directly address the situation and work towards better implementation for the subsequent session.

Separate conferences (generally every two to three months) were held for job skills intervention facilitators to support facilitators in their development. All facilitators were required to attend these conferences to share their perspectives on the program and also receive collective feedback on overall implementation. Conferences allowed for teambuilding and training. Lastly, extra source materials such as videos, websites, articles, and other media were consistently provided or recommended to support development of facilitator knowledge and skills and keep content up to date.

### 2.9. Outcomes

All outcomes are collected via self-report on anonymous surveys by participants (Table 2).

### 2.10. Primary outcome

#### 2.10.1. SV/ARA perpetration

At baseline and T3 (9 months post-intervention), participants report whether they perpetrated the following SV or ARA behaviors in the last 9 months. The primary outcome measure for the study is any report of SV or ARA perpetration, i.e., yes to any of the following items.

The following two constructs focus on ARA behaviors against a dating partner. “Any ARA perpetration” is measured as a yes to any of these 13 items.

#### 2.10.2. Physical/sexual relationship violence

Three items are used to assess physical or sexual violence perpetration against a partner or ex-partner [79]. Participants report whether they performed each action, which is dichotomized as yes to any. Examples include “hit, pushed, slapped, choked or otherwise physically hurt someone you were going out with or hooking up with?” and “used physical force or threats to make someone you were going out with or hooking up with have sex (vaginal, oral, or anal sex) when they didn’t want to?”

#### 2.10.3. Dating abuse

A ten-item scale, developed for use with high school-aged students, is used to assess whether the participant perpetrated any abuse against a dating partner [37]. Examples include “convinced them to have sex, after they said no a few times” and “told them not to talk to others or told them who they could hang out with. ” A positive response to any of these items is counted as any ARA perpetration.

“Any SV perpetration” is measured as yes to any of the sexual relationship violence and dating abuse items above as well as yes to any of the behaviors below.

#### 2.10.4. Non-partner sexual violence

To measure whether a participant committed sexual violence against a *non-partner,* the two sexual IPV items were modified to query for people they had NOT gone out with or hooked up with, and included friends, family, and strangers [79]. Responses are dichotomized as yes to any.

#### 2.10.5. Incapacitated sex

Participants are asked if they had done something sexual with someone when that person was “too drunk or high to stop you,” [80] with a response of “yes” coded as yes to incapacitated sex.

#### 2.10.6. Use of drugs or alcohol on purpose for sex

Participants are asked whether they had purposely given someone alcohol or drugs to do something sexual with that person [81]. A response of “yes” is coded as yes to use of drugs or alcohol on purpose for sex.

#### 2.10.7. Sexual harassment

Five items assess the frequency with which a participant has engaged in sexual harassment against someone from “making unwanted sexual comments” to “touching or grabbing them in a sexual way.” [82,83] Any endorsement of these behaviors is coded as yes for sexual harassment.

#### 2.10.8. Cyber sexual abuse

Given the ubiquity of social media and smartphones, three items assess for frequency of sexual harassment, including “try to get them to talk about sex when they did not want to” and “post or publicly share a nude or semi-nude picture of them” using mobile apps, social networks, texts, or other digital communication [3,84,85]. Any endorsement is coded as yes for cyber sexual abuse.

### 2.11. Secondary outcomes

#### 2.11.1. Positive bystander intervention behaviors

A scale developed for use with high school students is used to determine whether participants will intervene or interrupt in a positive manner when they witness disrespectful or abusive behaviors by peers [37]. The scale first assesses whether participants had witnessed nine different abusive behaviors among their peers (e.g., “making rude or disrespectful comments about a girl’s body, clothing, or make-up”). For each behavior witnessed, participants are asked if they performed three positive behaviors (e.g., “I talked to an important adult about it”). Reporting at least one positive response per behavior witnessed is summed for the 9 items, creating a maximum summary score equal to 9.

#### 2.11.2. Condom negotiation self-efficacy

A 5-item scale is used to assess how confident participants feel about negotiating condom use with a partner [86]. Three positive (e.g., “I feel confident in my ability to suggest using condoms with a new partner”) and two negative (e.g., “If I were unsure of my partner’s feelings about using condoms, I would not ask my partner to use one”; reverse coded) are used. Responses are on a 5-point Likert scale, with values from “strongly disagree” to “strongly agree.” The scale is analyzed using the mean score.

#### 2.11.3. Attitudes related to condom and contraceptive use

A 10-item scale evaluating the participants’ temperament towards the usage of condoms and other contraceptive modalities. Examples of these 10 items include [87–90], “using birth control makes sex feel unnatural” and “I am in favor of my partner and me using birth control. The attitudes are measured by utilizing a 5-point Likert scale from “strongly disagree” to “strongly agree” and a mean score is calculated; a higher score indicates a more positive attitude towards condom and contraceptive use.

#### 2.11.4. Recognition of ARA

Recognition of abusive behaviors is measured with a 12-item scale that addresses the ability of participants to recognize offensive and harmful actions against a partner as abusive [91]; for example, “name calling or insulting them” and “threatening to hit them”. Responses range on a 5-point Likert scale from “strongly disagree” to “strongly agree. ” A mean score across the 12 items is calculated, with the higher score indicating higher recognition of abusive behaviors.

#### 2.11.5. Gender equitable attitudes

A 13-item scale is utilized to measure participants’ views on gender-equitable norms [37,92,93], such as, “A guy never needs to hit another guy to get respect” and “I would be friends with a guy who is gay”. Response options are on a 5-point Likert scale from “strongly disagree” to “strongly agree” and a mean score across the 13 items is calculated. A higher mean is indicative of more equitable attitudes.

#### 2.11.6. Intentions to intervene with peers

Utilizing eight items, this attitudinal measure assesses the likelihood for a participant to intervene when witnessing a range of harmful behaviors amongst male peer students, similar to the scenarios for assessing actual bystander intervention behaviors described above [37]. Responses are on a 5-point Likert scale from “very unlikely” to “very likely” and a mean score across the eight items is calculated; a higher score indicates greater intentions to intervene.

### 2.12. Sample size

Power and sample size calculations were based on clinically meaningful differences between treatment groups with respect to changes in the outcomes across time (i.e. intervention effect). For the primary outcome - any SV/ARA perpetration at Time 3, the detectable difference between arms was calculated based on traditional methods that assumed a fixed number of clusters as well as fixed number of subjects per cluster [94]. Twenty-one clusters were randomized, assuming a within-cluster intra-class correlation (ICC) of 0.01 (within-school correlations for abuse perpetration, similar to our team’s prior work with a related sexual violence prevention program with male athletes [37,95]), and a 20% baseline SV perpetration rate in the control arm. With 866 participants (approximately 41 boys at each site) and an 80% retention rate at Time 3, we expect to have 80% power to detect an absolute difference in SV perpetration of 8.3 (42% relative decrease) due to the intervention. We anticipate having ample power to detect clinically meaningful changes in bystander behaviors and the secondary outcomes as well. Based on previous studies, the within-cluster ICCs for each of our secondary outcomes ranged from 0.006 to 0.01. If we assume the upper end of that range, we will have at least 80% power to detect standardized mean differences as small as 0.23 between study arms.

### 2.13. Recruitment

For recruitment of eligible youth, we relied on the network of community partners we had identified in the asset maps created for each neighborhood cluster. This included site leaders, program facilitators, recruiters with strong connections to their community, prevention specialists embedded in schools, school districts that offer community-based programs as an alternative to suspension, and the Community Intensive Supervision Program (CISP) for youth involved in the juvenile justice system. Participant recruitment started in July 2015 and continued until the sample size met our estimates, through May 2017.

Using respondent driven sampling (RDS), former and current participants could refer their friends to the program. Participants interested in RDS received a packet of information on how to recruit a friend or neighbor and five recruitment coupons. When newly-recruited participants came to their first session and turned in their recruitment coupon, the peer recruiter was compensated $5, up to $25 overall.

### 2.14. Assent and consent

Adolescent males ages 13–19 received a description about the research study and parental letter about the study from the community sites. The parent letter included an option for parents/caregiver to decline their child’s participation. We received a waiver of parental permission and waiver of signed consent from the University of Pittsburgh Human Subjects Research Protection Office. Research assistants reviewed the verbal consent form with youth at the beginning of the first session and answered any questions pertaining to confidentiality, the program flow, and survey time points. The consent form covered all 3 waves of data collection, Time 1 through Time 3 as described earlier.

### 2.15. Retention in program

Once community partners recruit youth, retention throughout the 18-h curriculum is a key focus. Upon enrolling in either the control or experimental arm, prospective participants received program information, signed an assent form and completed a contact information sheet. Research assistants ensure that the document is legible and that all fields are completed. Research assistants use this information to contact the participant prior to each session to remind them of the session. If youth are not present at the beginning of a session, research assistants and facilitators will contact the participant to encourage them to join late.

### 2.16. Assignment of interventions

#### 2.16.1. Randomization

Randomization was performed at the neighborhood level (i.e., cluster) to reduce risk for contamination. The initial randomization included 10 clusters that were assigned to experimental or control conditions. The study statistician performed this randomization, stratifying by lead site in that neighborhood (YMCA, Urban League, or Other), such that within each stratum, each site/neighborhood had a 50/50 chance of being assigned to intervention or control. Due to a combination of lower-than-expected recruitment by neighborhood (target = 96 participants each) and the interest from other community partners, we individually randomized an additional 11 neighborhoods, stratified by type of site. This resulted in a total of 21 clusters randomized, with 11 assigned to experimental and 10 assigned to control. All neighborhoods in the study met the original criteria of being socially or economically disadvantaged and/or predominantly African American.

#### 2.16.2. Blinding

Randomization was performed after approval for the study was obtained for a site in a new neighborhood so that the randomization assignment would not influence a site’s willingness to participate. Notably, the PI was blinded from randomization until she had successfully recruited a site to participate in the study. Due to the study design, investigators, research staff, community partners, facilitators, and youth participants could not be blinded to study assignment.

### 2.17. Data collection, management, and analysis

#### 2.17.1. Data collection

There are several points of data collection throughout the study, starting at baseline all the way through T3: baseline surveys, feedback forms, End of Program (EOP) survey, T2 and T3 follow up surveys. The surveys are all anonymous, linked by a personal study code that youth create by answering a series of questions that only they know the answer to at the beginning of each survey. This method of using a personal study code was selected to ensure anonymity and increase the likelihood of honest responses. [96–99]

In addition to survey data, other sources of data for this study (primarily for process evaluation and assessing intervention fidelity) include: 1) feedback forms completed by youth after the end of each session; 2) fidelity forms completed by research assistants at each session; 3) interviews with site leads and facilitators; 4) confidential interviews with youth (after Time 3 data collection).

#### 2.17.2. Data management

Baseline and follow-up survey participation coincided with the beginning of the intervention (Time 1), end of the program (EOP), and three (T2) and nine (T3) months following the end of the 18 h program (i.e., round). All sites conduct web-based surveys (back up paper surveys are used as needed) on tablets using REDCap, an online data management and survey system. Participation in the 3- and 9-month follow-up surveys (T2 and T3 surveys, respectively) are facilitated via tracking of participants with the help of community partners at each site. Youth provide detailed contact information at baseline to facilitate follow up. Contact information is confirmed again at sessions following the baseline survey, and at the T2 survey. Youth are also called or texted periodically by research assistants between follow-up surveys to ensure that contact information is still valid. Finally, for those that miss an EOP or T2 survey in the appropriate time frame, a comprehensive “make-up” survey is offered (with the same monetary compensation) to update contact information and increase the likelihood they’ll participate in the next survey.

Responses to the anonymous web-based secure survey are entered by the youth participants themselves on an electronic tablet; no data are stored on the computers themselves. Only research staff who have been added to the project can access this online database. Data are downloaded and stored on a password-protected share drive that can only be accessed by users with the appropriate permissions. No names are connected to the survey data as each participant creates their own secret code as described above.

The only study documents that contain unique personal identifiers are contact forms and the contact list of participants (youth and prevention educators) that are kept to assist with re-contacting participants for follow up surveys). Contact forms are stored in a secure file drawer inside the locked office of the PI whenever not in use. Contact forms are stored separately from any survey data collected in this study (the survey data are collected via computer and immediately housed in a password-protected secure database). The names of participants are kept in encrypted files on a password-protected server behind the UPMC firewall.

#### 2.17.3. Process evaluation data collection

Data are collected to assess the quality of program implementation. Research assistants are present at each intervention session and complete a fidelity form to ensure consistent implementation of the intervention or control program as intended as well as unforeseen barriers to implementation. These feedback forms are reviewed by the lead facilitators and PI regularly to provide immediate feedback to facilitators should mid-point corrections be needed.

Youth also complete a feedback form at the end of each session which is reviewed by the facilitators and research assistant to gauge youth interest and engagement in the topics and to make mid-point adjustments to program content and delivery as needed.

In addition to the end of program survey that encourages youth to provide feedback on the entire 18-h curriculum, after completion of the T3 (final) follow up survey, youth in the intervention arm are invited to participate in a semi-structured interview about their experiences with the program. Interviews with site leads and facilitators provide additional feedback on the program to guide ongoing implementation including sustainability in the participating neighborhoods. Collectively, process evaluation data will be used to inform and improve the intervention content and implementation guidance.

### 2.18. Statistical methods

Generalized linear mixed models will be used to account for the correlation among youth from the same cluster as well as the correlation between observations from the same youth. Descriptive statistics will be used to summarize the sample with regard to baseline characteristics of interest. Means and standard deviations will be presented for continuous variables, while sample proportions will be provided for categorical variables. 95% confidence intervals will accompany all sample statistics. Primary assessment of intervention effects will be based on intent-to-treat estimates. As-treated, or treatment-on-the-treated (TOT), effect parameters will be estimated in secondary analyses and reported as exploratory. Between-site differences regarding intervention effects will be assessed based on level of staff/facilitator engagement in curricular delivery as well as other observed external factors that may interact with the intervention to alter outcomes. SAS software will be used for all statistical analyses.

To assess differences at baseline between the youth in the experimental and control groups, demographics such as grade-level, race, nativity, and parental education will be compared while accounting for within-neighborhood clustering. Demographic variables as well as neighborhood-level characteristics resulting in between-arm imbalances will be considered as covariates in the primary and secondary analyses.

Participation bias will be assessed by comparison of age and race/ethnicity of youth participating in the study compared to the overall demographics of adolescents (school district and census data). Significant differences detected via chi-square analysis will be noted as potential validity threats.

An attrition analysis will be conducted by comparing youth who completed follow-up surveys with those who did not with regard to demographics as well as outcomes measured at baseline. All hypotheses will be two-sided tests with a significance level of 5%.

Missing data were minimized as much as possible by keeping the surveys as short as possible to reduce survey burden, encouraging youth to be as complete and honest as possible by ensuring anonymity of the surveys, and working assiduously with community partners to ensure that youth stay engaged and can be tracked to complete follow up surveys. Mechanisms for missing data will be investigated by comparing important covariates between youth with and without missing data at each time point. We will characterize these mechanisms as 1) missing completely at random (MCAR), 2) missing at random (MAR), or 3) not missing at random (NMAR). If the nature of our missing data is ignorable (either MCAR or MAR), our primary analysis will be sufficient given that it is a likelihood-based approach to handling missing data. Additionally, we will conduct various sensitivity analyses such as multiple imputation via chained equations (MICE) and complete-case analyses. If our missing data is non-ignorable (MCAR), we will investigate other methods such as joint or shared parameter models.

The primary outcome for this study is reductions in self-reported perpetration of SV and ARA at Time 3, compared to controls. We will examine whether Manhood 2.0 results in improvements in the primary outcome assessed at 9 months after end of program (Time 3) compared to controls. Generalized linear mixed models (GzLMM) will be used and will include variables for baseline SV/ARA perpetration, treatment group, and random effects for cluster. For the primary outcome and other binary outcomes, the GzLMMs will be fit using a logit link; for all other continuous outcomes, an identity link function will be used.

For positive bystander intervention and the remaining secondary outcomes, the models will include variables for the secondary outcome at baseline, treatment group, and random effects for cluster. This will allow us to assess the effect of the study arm after accounting for clustering within neighborhoods. As an exploratory analysis, all three time points will be included in a single generalized linear mixed model to quantify the long-term trajectories of each of the secondary outcomes.

Following Twisk and Proper’s recommendations for randomized controlled trials with both baseline and follow up measures (in this case abuse perpetration) [100], we will also construct multinomial logistic regression models that account for presence or absence of baseline perpetration. That is, we will examine intervention effects among youth reporting baseline SV/ARA perpetration and the likelihood of becoming ‘non abusive’ at follow up (i.e., an ‘early intervention’ effect). Similarly, we will examine intervention effects among youth with no baseline SV/ARA perpetration and the likelihood of ‘staying non abusive’ (i.e., a primary prevention effect). We have used this approach to analyze intervention effects on abuse exposure in our school health center relationship abuse prevention study [101].

We will also conduct two sets of exploratory analyses. First, we will conduct an intensity-adjusted analysis that reflects the actual delivery of the program. To achieve this, we will use a continuous score of “intensity” to replace the binary intervention variable in the models for the primary analysis. This score will be calculated for each round (program delivery) by using two sources of information. The first is information collected systematically about each session by trained research assistants who observed the sessions, including whether each task assigned to a module was performed (yes or no). The second is a summary measure of overall attendance for each round, tracked by using sign-in sheets with the research assistants. The second set will be a per-protocol analysis. This set of models will include only participants in the intervention arm that received the full intervention, defined as having covered a minimum threshold of tasks across the six sessions and having sufficient attendance. These measures replicate the intensity score values, but are then dichotomized to yes or no to receiving the full intervention. All control arm participants will be included in these models.

To explore whether demographics, youth pre-intervention risk and protective factors (e.g., history of SV/ARA exposure, sexual risk, connectedness), and site-level differences (e.g., staff experience, organizational capacity, intervention intensity) moderate the effect of the intervention on the primary and secondary outcomes, linear mixed models slightly different from those described above will be utilized. The outcome variables will be modeled as a function of the following variables: the outcome at baseline, the treatment group, the potential moderator, the interaction between the treatment group and moderator, and a random effect for cluster. A significant interaction suggests the presence of intervention effect heterogeneity, and we will follow the approach of Kraemer [102] by focusing on effect size derivation rather than formal hypothesis testing.

### 2.19. Monitoring

#### 2.19.1. Data monitoring

Given the sensitivity of the questions being asked regarding violence perpetration, we received a Certificate of Confidentiality from the Centers for Disease Control and Prevention to protect the research data from subpoena. Extra precautionary measures were taken to protect the data, including the use of a personally created ID code to maintain anonymity of the survey data and an internal data safety and monitoring plan, which included the following:

a) Systematically review assessment materials to ensure that assessment is conducted appropriately and that participants disclosing abuse or violence during the course of taking the survey receive appropriate connection to violence-related services and that mandated reports are made by site personnel when appropriate.
b) Systematically review notes from research assistants to ensure that participants experiencing distress are being connected directly with the site directors and youth workers, receiving educational materials, and being referred appropriately; this includes ensuring that all research assistants document asking each participant about emotional distress after completion of the survey.
c) Monitor staff performance with regard to protection of privacy, confidentiality, maintenance of secure data bases, and study procedures designed to reduce the risk of distress and potential breaches of confidentiality.
d) Ensure that the PI (Miller), or a designated qualified individual, will be available by pager in case research staff needs to confer regarding participants’ behaviors or comments made during a survey or other research activities.
e) Ensure that the PI (Miller), or a designated qualified individual, will be available by pager in case educators or violence prevention advocates from Center for Victims and Pittsburgh Action Against Rape, needs to confer regarding participants’ or youth workers’ behaviors or comments made during study implementation (i.e., during training, survey administration, or follow up contact with site administrators, youth workers and facilitators).
f) Review and report any adverse events associated with the study.

## 3. Results

**Fig. 3.**
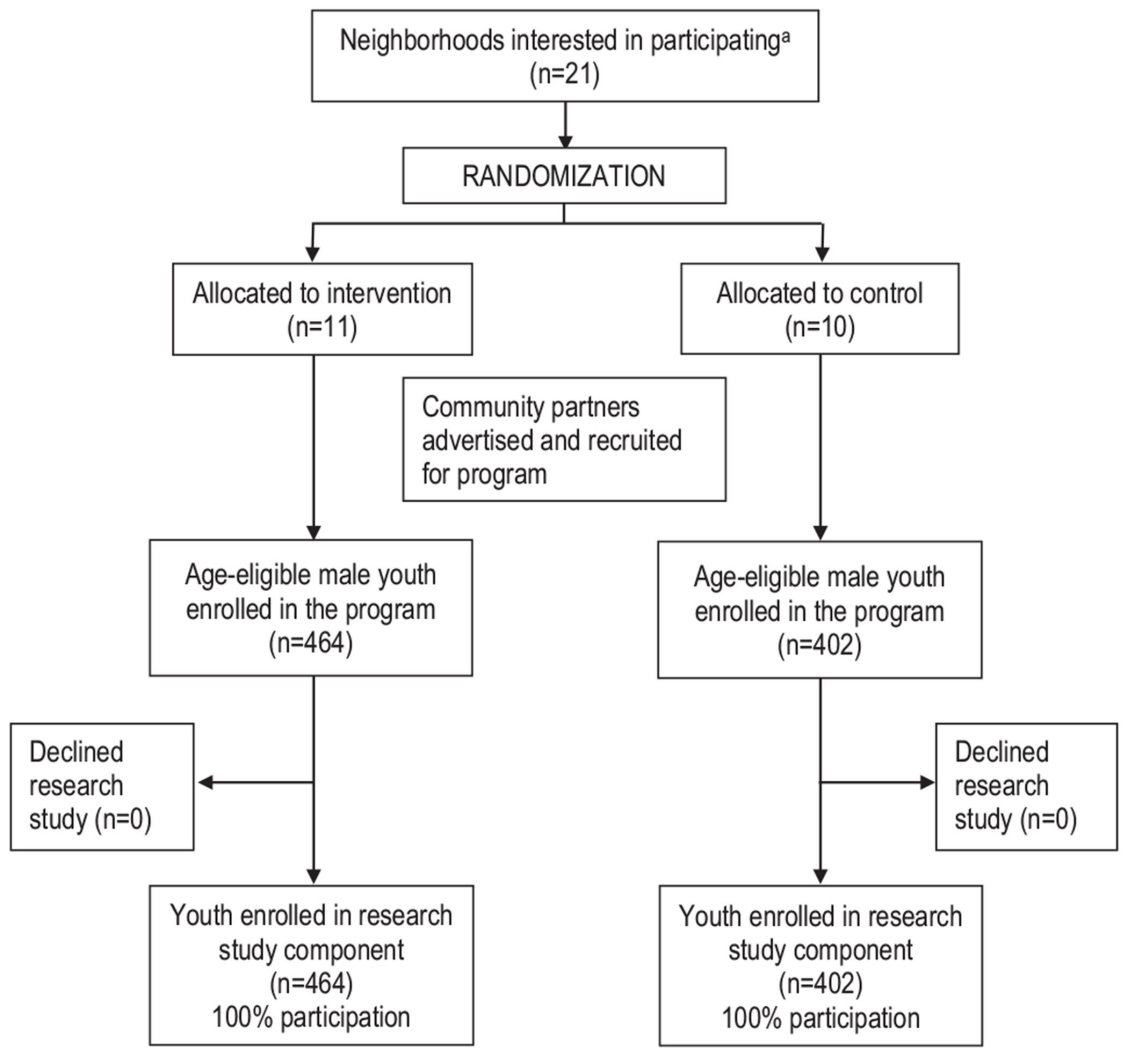
CONSORT diagram.

Related to recruitment, at least one site from the 21 neighborhoods agreed to participate (Fig. 3). Eleven neighborhoods were allocated to the experimental condition and ten to the control condition, and the neighborhood characteristics were similar between intervention and control neighborhoods (Table 3). There were a total of 866 age-eligible male youth who attended the program (464 in the experimental group; 402 in the control); all 866 enrolled in the study and completed a baseline survey (100% participation).

As detailed in Table 3, most participants (70%) identify themselves as Black or African American and 88% were born in the US. Participants’ age ranges overall were roughly evenly divided across 13–14, 15–16, and 17–19; the intervention arm had a slightly lower proportion of 13–14 year olds and higher proportion of 15–16 year olds compared to the control arm. At the time of their baseline survey, 22% were in middle school and 62% were in high school.

Compared to the control arm, the intervention group had higher proportions of those who self-identified as White, Multiracial, or Other racial category, and lower proportions of those who self-identified as Black/African American or Hispanic. Intervention and control arm participants were similar in all other characteristics.

## 4. Discussion

“Manhood 2.0” is a gender transformative curriculum adapted from the international setting that involves critical reflections, challenging of, and ultimately transforming, harmful gender and sexuality norms that foster violence against women and seeks to promote bystander intervention (i.e., giving boys skills to interrupt abusive behaviors they witness among peers) to reduce the perpetration of sexual violence (SV) and adolescent relationship abuse (ARA). This is a community-based cluster-randomized controlled trial in lower resource, Pittsburgh neighborhoods that involves high school age adolescent males to test the effectiveness of Manhood 2.0. The comparison intervention is a job readiness training program which focuses on skills needed to prepare youth for entering the workforce, including goal setting, accountability, resume building, and interview preparation. The primary outcome of interest is whether Manhood 2.0 reduces SV/ARA perpetration at Time 3 (9 months after program). Increases in positive bystander behaviors (i.e., intervening in a peer’s disrespectful or harmful behavior) is a secondary outcome. Other related intermediate outcomes are changes in recognition of what constitutes abusive behavior, intentions to intervene, and gender equitable attitudes.

Strengths of this study are the rigorous approach using a cluster-randomized controlled trial design combined with strong partnerships with multiple community partners including community leaders, youth serving agencies, churches, libraries, and school districts who facilitate recruitment and retention. Additionally, close attention to fidelity to intervention will allow for exploratory analyses about implementation: organizational and facilitator-level characteristics that contribute to high fidelity to the intervention; strategies community facilitators use for introducing and facilitating discussions; and barriers and facilitators for intervention implementation.

The study also has several limitations. The surveys are collected anonymously with each participant creating their own personal identification code that only they will know to match surveys over time. Thus, the dosage of program received (i.e., proportion of program completed) can only be calculated at the level of each round rather than at the individual level. As a community-based study recruiting youth who are at high risk for school ‘push out,’ retention of this cohort remains a critical challenge, with the most vulnerable at especially high risk for being lost to follow up. Additionally, youth who are juvenile justice system-involved are particularly difficult to retain. Thus, we are likely to have significant missing data from rounds involving justice system-involved youth. We will conduct sensitivity analyses both with and without these youth in the sample to examine differences in intervention effects.

In summary, this study protocol is intended to evaluate Manhood 2.0 using a rigorous design to determine the effectiveness of a community-based sexual violence and adolescent relationship abuse prevention program for high school age youth living in low resource neighborhoods. Findings may provide urgently needed information about the effectiveness of a gender transformative program that combines healthy sexuality skills, gender norms change, and bystander skills to interrupt peers’ disrespectful and harmful behaviors to reduce SV/ARA perpetration among adolescent males.

## Figures and Tables

**Table 1:** Neighborhood characteristics.

Neighborhood	Non-white students	Economically Disadvantaged^a^	Student stability rate^b^	Graduation rate^c^	Chronic Absenteeism^d^	School Suspension^e^
Control (median)	84%	80%	81%	81%	56%	30%
Allentown/Beltzhoover/Hilltop*	45%	66%	83%	86%	43%	23%
Clairton^†^	81%	99%	n.a.	79%	n.a.	n.a.
Garfield*	95%	81%	81%	81%	56%	31%
Hazelwood*	52%	40%	88%	88%	28%	12%
Hill District*	95%	81%	81%	81%	56%	31%
North Braddock^†^	65%	100%	n.a.	83%	n.a.	n.a.
Northside*	87%	79%	70%	72%	69%	30%
Northview*	87%	79%	70%	72%	69%	30%
Downtown*	64%	67%	88%	92%	13%	23%
Wilkinsburg*	99%	80%	72%	77%	66%	36%
**Intervention (median)**	**52%**	**64%**	**72%**	**84%**	**66%**	**36%**
Duquesne^†^	35%	45%	n.a.	93%	n.a.	n.a.
East Hills*	52%	40%	88%	88%	28%	12%
East Liberty*	99%	80%	72%	77%	66%	36%
Homewood*	99%	80%	72%	77%	66%	36%
Larimer*	99%	80%	72%	77%	66%	36%
McKeesport^†^	51%	100%	n.a.	85%	n.a.	n.a.
Munhall^†^	38%	56%	n.a.	97%	n.a.	n.a.
Penn Hills^†^	67%	61%	n.a.	84%	n.a.	n.a.
Prospect Park^†`^	16%	33%	n.a.	94%	n.a.	n.a.
Sheraden*	64%	64%	85%	78%	36%	17%
Wilmerding^†^	41%	100%	n.a.	79%	n.a.	n.a.
Kolmogorov-Smirnov two-sample exact test comparing intervention and control, *p*-value	0.3230	0.1088	0.8695	0.5783	0.8695	0.2945

n.a. indicates data were not available for the neighborhood school district.

* Reference [103].

† Reference [104].

a Economically disadvantaged: Students are identified as economically disadvantaged based on the state’s Direct Certification process, which can include poverty data sources such as the Supplemental Nutrition Assistance Program, Transitional Assistance for Families with Dependent Children, or Medicaid eligibility; and children living in foster care.

b Student stability rate: total number of students who didn’t transfer during the entire year divided by the official enrollment for that year, which is calculated in October.

c Graduation rate: rate of individual 9th graders in 2012 who graduated in 2016 or earlier.

d Chronic absenteeism: the percentage of students who were absent 10% or more of the days they were enrolled in the school.

e School suspension: percentage of students who were suspended (out-of-school suspensions only) at least once during the school year.

**Table 2:** Individual youth-level outcome measures.

Construct	Response options	Surveys				Items
	Primary Outcome: Any Sexual Violence/Adolescent Relationship Abuse Perpetration (comparing differences between intervention and control at Time 3)					
Physical/sexual IPV [79]	Yes/no to each Modeled as yes to any lifetime (baseline only) and yes to any past 9 months	X			X	*Have YOU done any of the following to someone you were in a relationship with (like he or she was your partner/girlfriend/boyfriend, you were dating or going out with, them) or hooking up with:* 1. …[ever/in the past 9 months] hit, pushed, slapped, choked or otherwise physically hurt someone you were going out with or hooking up with? (include such things as hitting, slamming into something, or injuring with an object or weapon.) 2. …[ever/in the past 9 months] used physical force or threats to make someone you were going out with or hooking up with have sex (vaginal, oral, or anal sex) when they didn’t want to? 3. … [ever/in the past 9 months] had sex with someone you were going out with or hooking up with when they didn’t want to or because you made them feel like they didn’t have a choice (even though you did not use physical force or threats)?
Dating abuse [37]	Baseline - No, I have never done this to someone I was in a relationship with - Yes, I have done this in the past 9 months - Yes, I have done this, but not in the past 9 months T3: Yes/no Modeled as summary score for lifetime (baseline only) and past 9 months	X			X	*Have YOU done any of the following to someone you were going out with (like he or she was your partner/girlfriend/boyfriend, you were dating them) or hooking up with:* 1. Spread rumors about their sexual reputation, like telling people they’re ‘easy’. 2. Convinced them to have sex, after they had said no a few times. 3. Made them have sex when they didn’t want to. 4. Physically hurt them (like shoving, grabbing, slapping, punching, choking). 5. Threatened to hurt them if they didn’t do what you wanted them to do. 6. Yelled at them or destroyed something that belonged to them. 7. Called them names, like ugly or stupid. 8. Told them not to talk to others or told them who they could hang out with. 9. Showed friends or posted pictures of them naked or doing something sexual. 10. Talked about what you and your partner do sexually with your friends or peers.
Non-partner sexual violence [79]	Yes/no to each Modeled as yes to any lifetime (baseline only) and yes to any past 9 months	X			X	*Now think about experiences you may have had with people who you were NOT going out with or hooking up with (this could include strangers, friends, family, or people you don’ know well).* *Please tell us whether YOU have [ever/in the past 9 months] done these things to anyone you were NOT going out or hooking up with:* 1. …used physical force or threats to make someone you were not going out with or hooking up with have sex (vaginal, oral, or anal sex) with you when they didn’t want to? 2. …insisted that someone you were not going out with or hooking up with have sex (vaginal, oral, or anal sex) when they didn’t want to, without using force or threats?
Sexual harassment [82,83]	4 point scale for frequency: never/a few times/once or twice a week/every day or almost every day Modeled as a summary score of responses of “a few times” or more often	X			X	*In the past 9 months since [insert month], how often have you done the following things to someone when they did not want you to?* 1. Made unwelcome sexual comments, jokes, gestures, or looks? 2. Showed, gave, or left sexual pictures, drawings, messages, or notes? 3. Spread sexual rumors about them? 4. Touched, grabbed, or pinched them in a sexual way? 5. Forced them to kiss you?
Cyber sexual abuse [3,84,85]	4 point scale for frequency: never/a few times/once or twice a week/every day or almost every day Modeled as yes for any response of “a few times” or more often	X			X	*In the past 9 months since [insert month], how often did you do the following to someone? How often did you…* 1. …try to get them to talk about sex when they did not want to… 2. …ask them to do something sexual that they did not want to do… 3. …post or publicly share a nude or semi-nude picture of them… …using mobile apps, social networks, texts, or other digital communication?
Incapacit-ated sex [80]	Yes/no	X			X	In the past 9 months since [insert month], have you done something sexual with someone when they were too drunk or high to stop you (this can include kissing, touching, fingering them, or having intercourse)?
Use of alcohol or drugs on purpose [81]	Yes/no	X			X	In the past 9 months since [insert month], have you given someone alcohol or drugs on purpose so you could do something sexual with them (this can include kissing, touching, fingering them, or having intercourse)?
		**T1**	**EOP**	**T2**	**T3**	
	**Secondary Outcomes: bystander behaviors, intentions to intervene, gender attitudes, recognition of abusive behavior, condom and contraceptive self-efficacy (differences between intervention and control at Time 2 and Time 3)**					
Positive bystander intervention behaviors [37]	I have not experienced this in the past 3 months. (0) I didn’t say anything. (−1) I told the person in public that acting like that was not okay. (+1) I laughed or went along with it (−1) I told the person in private that acting like that was not okay. (+1) I talked to an important adult about it privately (like youth leader, teacher, coach). (+1) Sum items with at least 1 positive response behavior (noted as +1) endorsed	X		X	X	*The following questions ask about specific behaviors that you may have seen or heard among your male peers or friends. If you experienced this at least once in the past 3 months, how did you respond?* 1. Making rude or disrespectful comments about a girl’s body, clothing, or make-up. 2. Spreading rumors about a girl’s sexual reputation, like saying “she’s easy.” 3. Telling sexual jokes that disrespect women and girls. 4. Bragging about what they and their girlfriend do sexually. 5. Showing other people sexual messages or naked/sexual pictures of a girl on a cell phone or the internet. 6. Doing unwelcome or uninvited things toward a girl (or group of girls) such as howling, whistling, or making sexual gestures. 7. Fighting with a girl where he’s starting to cuss at or threaten her. 8. Taking sexual advantage of a girl (like touching, kissing, having sex with) who is drunk. High from drugs, or passed out. 9. Shoving, grabbing, or otherwise physically hurting a girl.
Condom negotiation self-efficacy [86]	5 point Likert scale from ‘strongly disagree’ to ‘strongly agree,’ modeled as a mean score	X		X	X	1. I feel confident in my ability to discuss condom use with any partner I might have. 2. I feel confident in my ability to suggest using condoms with a new partner. 3. If I were to ask my partner to use a condom, I would be afraid that my partner would be upset with me. *(reverse coded)* 4. If I were unsure of my partner’s feelings about using condoms, I would not ask my partner to use one. *(reverse coded)* 5. If my partner didn’t want to use a condom during sex, I feel confident in my ability to refuse to have sex.
Attitudes related to condom and contraceptive use [87–90]	5 point Likert scale from ‘strongly disagree’ to ‘strongly agree,’ modeled as a mean score	X		X	X	*Please rate the following statements from Strongly Disagree to Strongly Agree:* 1. Using birth control keeps your partner from worrying about getting pregnant. 2. Using birth control gives you a sense of control. 3. Using birth control is too much trouble or too much of a hassle to use. 4. Using birth control takes too much planning ahead of time to have birth control on hand when you’re going to have sex. 5. Using birth control lets you have sex without worrying about getting your partner pregnant. 6. Using birth control makes sex feel unnatural. 7. Using birth control makes sex less exciting. 8. It does not matter whether you use birth control or not; when it’s your time to get pregnant, it will happen. 9. It is mainly a girl or woman’s responsibility to make decisions about birth control. 10. I am in favor of my partner and me using birth control.
Recognition of ARA [91]	5 point Likert scale from ‘not abusive’ to ‘extremely abusive,’ modeled as a mean score	X		X	X	*Please rate each of the following actions towards a girlfriend or boyfriend as not abusive, a little abusive, somewhat abusive, very abusive or extremely abusive.* 1. Name calling or insulting them. 2. Telling them they’re ugly or stupid. 3. Making fun of them in front of other people. 4. Telling them what to do all the time. 5. Telling them which friends they can and can’t see or talk to. 6. Pressuring them not to break up with them. 7. Not listening to what they have to say. 8. Trying to convince them to have sex. 9. Preventing them from leaving a room. 10. Keeping tabs on them or spying on them. 11. Threatening to hit them. 12. Forcing them to have sex.
Gender-equitable attitudes [37,92,93]^a^	5 point Likert scale from ‘strongly disagree’ to ‘strongly agree,’ modeled as a mean score	X		X	X	*Please rate the following statements from strongly disagree to strongly agree.* 1. A guy takes responsibility for his actions. 2. A guy never needs to hit another guy to get respect. 3. A girl wearing revealing clothing deserves to have comments made about her.^b^ 4. It bothers me when a guy acts like a girl.^b^ 5. Guys should sleep with as many girls as possible.^b^ 6. If a guy tells people his worries, he will look weak.^b^ 7. In a good dating relationship, the guy gets his way most of the time.^b^ 8. Guys should only have sex with girls.^b^ 9. I can respect a guy who backs down from a fight. 10. I would be friends with a guy who is gay. 11. A guy should share in household chores. 12. If a girl is raped it is often because she did not say “no” clearly enough.^b^ 13. Guys put women and children first.
Intentions to intervene with peers [37]	5 point Likert scale from ‘very unlikely’ to ‘very likely,’ modeled as a mean score	X		X	X	*How likely are YOU to do something to try and stop wha’s happening if a male friend or peer (someone your age) is:* 1. Making rude or disrespectful comments about a girl’s body, clothing or make-up. 2. Spreading rumors about a girl’s sexual reputation, like saying “she’s easy”. 3. Fighting with a girl where he’s starting to cuss at or threaten her. 4. Doing unwelcome or uninvited things toward a girl (or group of girls) such as howling, whistling or making sexual gestures. 5. Shoving, grabbing, or otherwise physically hurting a girl. 6. Showing other people sexual messages or naked/sexual pictures of a girl on a cell phone or the internet. 7. Telling sexual jokes that disrespect women and girls. 8. Taking sexual advantage of a girl (like touching, kissing, having sex with) who is drunk, high from drugs, or passed out.

ID, investigator-developed.

a Formative research was used to create this scale with NICHD R24HD080194.

b Items were reversed coded.

**Table 3:** Baseline characteristics of participants.

Demographics	Total (n=866) % (n)	Treatment arm		p-Value^a^
		Intervention (n = 464) % (n)	Control (n = 402) % (n)	
Age (years)				0.0707
13–14	32.3 (280)	28.5 (132)	36.8 (148)	
15–16	39.0 (338)	42.5 (197)	35.1 (141)	
17–19	28.4 (246)	28.9 (134)	27.9 (112)	
Race				0.0075
Black/African American	70.3 (609)	68.1 (316)	72.9 (293)	
White	3.4 (29)	4.7 (22)	1.7 (7)	
Hispanic	6.1 (53)	3.5 (16)	9.2 (37)	
Multiracial	6.4 (55)	8.0 (37)	4.5 (18)	
Other	8.1 (70)	10.3 (48)	5.5 (22)	
Born in the United States				0.6619
Yes	87.5 (758)	88.2 (409)	86.8 (349)	
No	5.7 (49)	6.7 (31)	4.5 (18)	
Education status				0.3406
Currently in school	84.8 (734)	85.8 (398)	83.6 (336)	
Not in school – completed high school degree	3.2 (28)	3.0 (14)	3.5 (14)	
Not in school – did not complete high school degree	4.9 (42)	4.1 (19)	5.7 (23)	
Current grade level^b^				0.3691
8th	22.2 (163)	18.8 (75)	26.2 (88)	
9th	24.5 (180)	25.1 (100)	23.8 (80)	
10th	20.4 (150)	21.9 (87)	18.8 (63)	
11th	17.7 (130)	18.8 (75)	16.4 (55)	
12th	9.8 (72)	11.1 (44)	8.3 (28)	
College	0.8 (6)	0.5 (2)	1.2 (4)	
Parents’/guardians’ highest education				0.7656
Did not complete high school	43.7 (378)	41.8 (194)	45.8 (184)	
Completed high school or GED	17.2 (149)	17.0 (79)	17.4 (70)	
Some college	7.6 (66)	7.5 (35)	7.7 (31)	
College degree or higher	24.0 (208)	25.9 (120)	21.9 (88)	

a Wald log-linear chi square, accounting for clustering.

b Among those who reported currently being in school.
